# Association and Occurrence of Bifidobacterial Phylotypes Between Breast Milk and Fecal Microbiomes in Mother–Infant Dyads During the First 2 Years of Life

**DOI:** 10.3389/fmicb.2021.669442

**Published:** 2021-06-07

**Authors:** Wenli Yan, Baolong Luo, Xuyao Zhang, Yongqing Ni, Fengwei Tian

**Affiliations:** ^1^School of Food Science and Technology, Shihezi University, Shihezi, China; ^2^School of Food Science and Technology, Jiangnan University, Wuxi, China

**Keywords:** human breast milk, mother–infant dyads, microbiome, *Bifidobacterium*, association

## Abstract

Breast milk acts as an intermediary for the transfer of functionally important commensal bacteria from mother to infant, especially for *Bifidobacterium* that can colonize the infant gut. However, the vast majority of rRNA amplicon-based studies reported the conspicuous intercohort and interindividual variation for the prevalence of *Bifidobacterium* in breast milk. In order to elucidate whether *Bifidobacterium* phylotypes persistently co-occured at the species or strain level in mother–breast milk–infant triads, we analyzed collectively the next-generation sequencing (NGS) datasets of bacterial 16S rRNA gene and the *Bifidobacterium*-specific *gro*EL gene from maternal feces, breast milk, and infant feces in a small yet very homogeneous cohort of 25 healthy Uyghur mother–infant pairs (lactation for 7–720 days) in Kashgar, Xinjiang, China. Overall, 16S rRNA gene analysis showed that microbiome in the newborn gut was closer to that of breast milk in the first 4 months of lactation, and subsequently showed an obvious trend of adulthood at 6–12 months. Based on the BLAST accurate taxonomic result of the representative sequences of all ASVs (amplicon sequencing variants), only three sets of ASVs could be clearly assigned into *Bifidobacterium* species, whereas the remaining eight sets of ASVs corresponded to four indefinite *Bifidobacterium* species group. By contrast, the *groEL* gene dataset was partitioned into 376 ASVs, at least belonging to 13 well-known *Bifidobacterium* species or subspecies, of which 15 ASVs, annotated to seven well-known *Bifidobacterium* species or subspecies, showed triadic synchronism in most 23 mother–infant pairs tested. However, several other rare bifidobacterial phylotypes, which were frequently encountered in animals, were found to display no correspondence of the presence between the three ecosystems of mother–infant pairs. Our test results were obviously to support the hypothesis that breast milk acts as an intermediary for the transfer of probiotic commensal bacteria from mother to infant, especially for endosymbiotic *Bifidobacterium* that can colonize the infant gut. Some oxygen-insensitive exogenous *Bifidobacterium* phylotypes with a cosmopolitan lifestyle may be indirectly transferred to breast milk and the infant’s intestinal tract through environmental contamination. Thus, the *gro*EL gene proved to be a very effective target for the depth resolution of *Bifidobacterium* community by high-throughput sequencing technologies.

## Introduction

Breast milk is generally regarded as the best source of nutrients for healthy growth and development of infants (Bode, 2012; Ballard and Morrow, 2013). Over the past decade and a half, many studies have focused on the bacterial community of breast milk using both culture-dependent and culture-independent techniques. Culture-independent techniques, based on the amplicon analysis of 16S rRNA gene, revealed the presence of several hundred bacterial species from breast milk up to now, the most common genus of which includes *Staphylococcus*, *Streptococcus*, *Flavobacterium*, *Propionibacterium*, *Burkholderia*, *Rothia*, *Corynebacterium*, and *Lactobacillus*, commonly known as a core milk microbiota (Heikkila and Saris, 2003; Groenlund et al., 2007; Martin et al., 2007; Perez et al., 2007; Collado et al., 2009; Hunt et al., 2011; Fitzstevens et al., 2017).

The origins of the bacteria in breast milk are thought to include the maternal gut (via an entero-mammary pathway) and through bacterial exposure of the breast during nursing (skin microbiota and the oral cavity of the infant) (Mueller et al., 2015). Increasingly, accumulating evidence highlights that the maternal gut serves as the most relevant source of bacteria that are detectable in breast milk. With the consumption of breast milk, and the reduced oxygen/redox potential in the infant gut, the obligate anaerobic bacteria emerge in the infant gut, especially the *Bifidobacterium* species and *Bacteroides* species (Ferretti et al., 2018). In the skin microbiome of a healthy human, by contrast, a member of *Bifidobacterium* was not detected (Oh et al., 2016), and was reported occasionally in the oral cavity of infants and the vagina of their mothers (Sundin et al., 2017; Dzidic et al., 2018; Nyangahu and Jaspan, 2019). So, breast milk constitutes the main source of an array of potentially beneficial bacteria to the breastfed infant gut.

Previous studies showed that total bacteria concentration is lower in colostrum than in transitional and mature milk, with increasing levels of *Bifidobacterium* in breast milks over lactation time (Cabrera-Rubio et al., 2012; Khodayar-Pardo et al., 2014; Sakwinska et al., 2016). Indeed, in a previous study, no bifidobacteria were detected from colostrum, and *Bifidobacterium s*trains were isolated only from breast milk samples obtained 7 days after birth or later. Again, despite the use of advanced next-generation sequencing (NGS) of 16S rRNA gene amplicon, presences of *Bifidobacterium* spp. were sporadically reported in a few colostrum samples or not at all. In contrast, the prevalence of bifidobacteria in transitional and mature milks was generally increased, but its relative abundance is only 0.1–1.3% or lower. More notably, *Bifidobacterium* populations were not also detected in a considerable proportion of transitional and mature milk samples, although an identical test method was applied to the same batch of samples (Hunt et al., 2011; Urbaniak et al., 2016; McGuire and McGuire, 2017; Murphy et al., 2017).

In general, bacterial loads of feces samples are five to seven orders of magnitude higher than in breast milk (Fernandez et al., 2013). From 1 to 6 months of age, members of the genus *Bifidobacterium* clearly dominate the infant gut microbiota, regardless of delivery mode, representing an average of 10–90% of the total infant gut microbiota (Turroni et al., 2012; Lim et al., 2016; Lundgren et al., 2018). However, a small proportion of infants have very low abundance or undetectable bifidobacteria as members of the fecal microbiota regardless of breast milk or formula feeding (Koenig et al., 2011; Yatsunenko et al., 2012; Subramanian et al., 2014).

While multiple studies have shown that specific *Bifidobacterium* strains in the maternal gut are transferred to the infant gut through breastfeeding (Makino et al., 2011, 2013; Milani et al., 2015; Duranti et al., 2017), the sources and ways of acquisition of these potential probiotic bacteria regarding establishing a sound intestinal microbiome for infants are poorly understood. As a whole, it is not clear how bifidobacterial community from the maternal gut or breast milk progressively transmits to the infant gut during the first 2 years of life, and whether there is the concordance of the presence of *Bifidobacterium* spp. between the three ecosystems, represented by maternal feces, breast milk, and neonatal feces.

Next-generation sequencing is a more sensitive and less biased analytical method than the culture-based method (Hunt et al., 2011; Jost et al., 2013; Ward et al., 2013). These methods generate tens of thousands of 16S rRNA gene sequences per DNA sample, but taxa present in very low abundance could still be missed (Meehan et al., 2018). In addition, 16S rRNA gene-based profiling of the human microbiota is strongly influenced by sample processing and the choice of PCR primers, leading to underrepresentation of bifidobacteria in 16S rRNA sequence dataset. Also, it is difficult to identify the members of *Bifidobacterium* at species level by 16S rRNA short variable region amplicon sequencing (Schloss and Westcott, 2011). All these reasons result in inconsistent detection of the proportional abundance of specific bacteria taxa of human microbiota, including *Bifidobacterium* spp. Particularly, breast milk samples are highly variable in bacterial load values. There are large individual differences over time between samples from the different mothers and, in some cases, even within individuals at different time points (Bode et al., 2014; Moossavi et al., 2019). Therefore, we are more interested in the co-occurrence and combination of gut symbiotic *Bifidobacterium* phylotypes in mother–breast milk–infant triads than in their absolute content.

More recently, the *groEL* gene proved to be a very effective target for the identification and quantification of *Bifidobacterium* spp. through high-throughput sequencing technologies or qPCR (Junick and Blaut, 2012; Hu et al., 2017). In this study, a comparative analysis of feces and breast milk microbiota in a small, yet very homogeneous cohort of 25 healthy mother–infant pairs in Kashgar, northwest China (*n* = 25, infants’ age from 7 days to 2 years), was presented, using high-throughput sequencing technologies of the 16S ribosomal RNA gene and *groEL* gene specific to the genus *Bifidobacterium*. Our research objective is to assess the association of bifidobacterial phylotypes between infant feces and their mothers’ breast milk and maternal feces in mother–breast milk–infant triads, with the emphasis on the number and changes of *Bifidobacterium* phylotypes in the infant feces and breast milk.

## Materials and Methods

### Sample Collection

We collected breast milk and feces samples from mothers and their infants between 7 and 720 days after birth, during clinic or home study visits and recruited mother–infant pairs meeting the following criteria: (i) the Uighur people native to Kashgar, Xingjiang, (ii) vaginal delivery at full-term (≥37 week gestation), (iii) exclusive breastfeeding during the first 5 months and the lactation continuing until sampling, and (iv) no antibiotic/probiotic exposure of either the mother or the infant during pregnancy, intrapartum, or postnatally. All the participants were healthy and did not require hospitalization. They were included for microbiota analysis with standard collection protocol (Sakwinska et al., 2016). Nevertheless, our samples were collected from all the mother–infant pairs for only one time, and a longitudinal study was not carried out.

Demographic and clinical data were recorded in a specific case report form. All participants responded to a general questionnaire including socioeconomic, lifestyle aspects, and body mass index (BMI) of the mother. The case report recorded the number of gestational weeks at delivery, delivery method, feeding patterns, the gender, height, weight, and head circumference (for the newborn) of the infant. All demographic data of our cohort is summarized in Table 1, and this cohort has been reported in a previous study, which was focused on *Lactobacillus* (Zhang et al., 2020).

**TABLE 1:** Demographic characteristics of our cohort.

**Characteristics and demographic data**	**Values or no. (%)**
**Infant gender**	
Male	15 (60)
Female	10 (40)
**Maternal BMI condition**	
Normal (18.5–23.9)	16 (64)
Slightly fat (24.0–26.9)	4 (16)
Obesity (27–29.9)	3 (12)
Severe obesity (≥30)	1 (4)
Unknown	1 (4)
**Infant age at specimen collection (days)**	
7–180	8 (32)
206–365	8 (32)
366–720	9 (36)
**Feeding patterns**	
Exclusively	9 (36)
Partial feeding	16 (64)
Infant weight (kg)	7.58 ± 2.89*
Infant length (cm)	58.50 ± 9.63*
Infant age (days)	312.04 ± 191.71

*Mean ± SD.

Standard sterile collection tubes were used to collect feces and breast milk (with the aid of a breast pump) samples. For breast milk, the first few drops (0.5–1 ml) were discarded, and the breast was thoroughly cleansed with chlorhexidine solution before manually collecting 3–5 ml of milk. Samples were immediately transported to the laboratory using portable refrigerators and ice packs. Each breast milk sample was divided into several 1-ml servings into sterile centrifuge tube and 500-mg feces sample were divided into a sterile centrifuge tube ready for DNA extraction and then were all frozen at −80°C in batches for processing and remained frozen until DNA extraction. All samples were collected and stored on the same day, and total bacterial DNA was extracted within 7 days of sampling as well as sequenced, to reduce the errors caused by the condition of storage, experiment, and sequencing.

### DNA Extraction and High-Throughput Sequencing

For breast milk samples, TIANamp Blood DNA Kit (TIANGEN, Beijing, China) was utilized with some modifications, referring to Sakwinska et al. (2016) to extract bacteria DNA. One milliliter of breast milk was centrifuged at full speed (12,000 rpm) for 10 min at 4°C. The pellets were resuspended in 200 μl of Tris-EDTA buffer and treated with 10 μl of lysozyme (50 mg/ml) and 5 μl of DNase-free RNase (20 mg/ml) for 30 min at 37°C. Twenty-five milligrams of glass beads (10 μm) was added to the solution and treated with three bead-beating steps in a FastPrep instrument (MP Biomedicals, Irvine, CA, United States) at 5.5 movements per second for 1 min. After the instantaneous centrifugation, the supernatants were collected and treated with 20 μl of proteinase K for 20 min at 56°C. Two hundred microliters of GB buffer was added, and samples were incubated at 65°C for 10 min, and then 200 μl of ethanol was added. DNA was further purified using Spin Columns CB3 (TIANGEN) following the manufacturer’s instructions. The feces samples were processed with the TIANamp DNA Stool Kit (TIANGEN, Beijing, China) according to the manufacturer’s instructions. Extracted DNA was quantified using the nucleic acid quantifier (NanoDrop Technologies, Wilmington, DE, United States).

### Sequencing Data Processing

For each sample, the V4–V5 region of the 16S rRNA gene and the *groEL* gene was amplified and sequenced according to the manufacturer’s instructions (Illumina, San Diego, CA, United States) by Shanghai Personal Biotechnology Co., Ltd., Shanghai, China^1^. Primer pairs for *groEL* sequences (Bif-groEL-F: 5-TCC GAT TAC GAY CGY GAG AAG CT-3/Bif-groEL-R: 5-CSG CYT CGG TSG TCA GGA ACA G-3) belonging to the target *Bifidobacterium* and available in GenBank release 234.0 (Benson et al., 2011) were designed by the Jiangnan University (Hu et al., 2017).

Raw sequences were processed by using a pipeline combining USEARCH v10.0 (Edgar, 2010) and QIIME (Caporaso et al., 2010). High-quality reads, as selected using the default values in USEARCH, were binned into ASVs (amplicon sequence variants) according to the denoising (error correcting) Illumina amplicon reads using Unoise3 (Edgar, 2017), through an open-reference strategy. Taxonomic identification of ASVs for the V4–V5 region sequences was assigned using the Naive Bayes classifier of the Ribosomal Database Project (RDP) against Greengenes database (August 2013 release). Meanwhile the taxonomy of ASVs for the *groEL* sequences was performed through comparison with the Chaperonin Sequence Database^2^ (Hill et al., 2004) and the National Center for Biotechnology Information (NCBI).

The diversity index was calculated by QIIME, and statistics and plot were performed using R software (version 4.0.2). Observed ASVs and Shannon index were analyzed as alpha rarefaction metrics. Weighted and unweighted UniFrac distances were computed as beta diversity, which was used for principal coordinate analyses (PCoA), and the function “adonis” of the *vegan* package of R software was utilized to test the significance of separation by permutational multivariate analysis of variance. *p*-values were corrected for multiple comparisons using the Benjamini–Hochberg method. *p* < 0.05 was considered statistically significant. The R function *hclust()* and package *ggtree* were utilized to cluster samples based on the Bray–Curtis similarity index using average linkage clustering and generate the dendrogram. Linear discriminant analysis (LDA) effect size (LEfSe) analysis was performed by R package *dplyr* and an open-reference strategy (Zhang et al., 2018). The bifidobacterial co-occurrence relationship of breast milk, infant feces, and maternal feces was determined based on the Spearman correlation coefficient and was visualized using the *AnnotationDBi* package.

## Results

### Microbial Community Structures in Breast Milk

**FIGURE 1:**
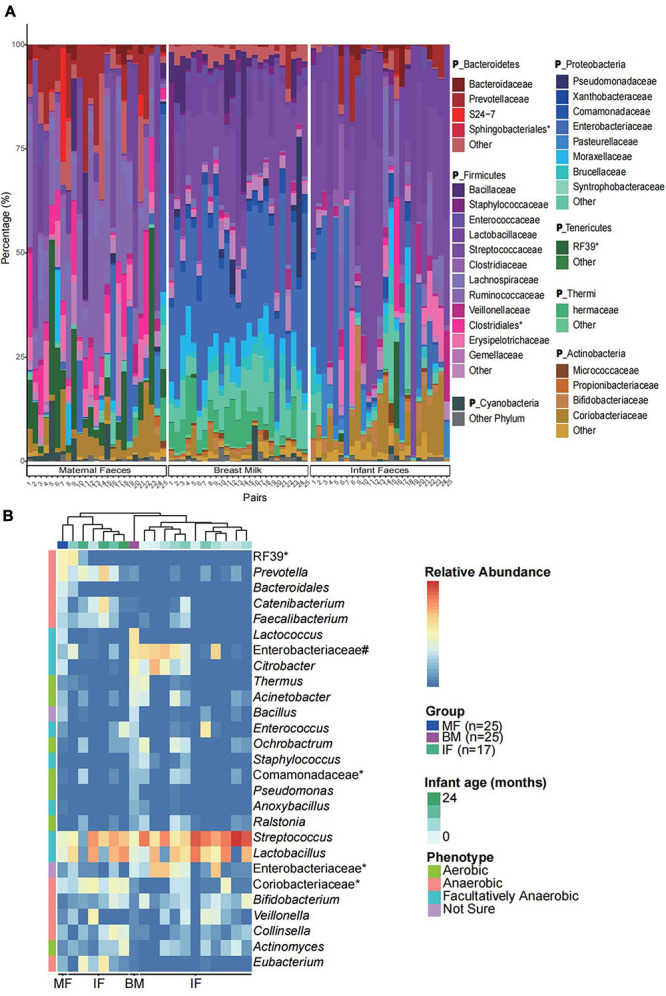
Microbial community characteristics of the infant feces, maternal feces, and breast milk samples. **(A)** Community structure of the dominant bacteria in breast milk and feces samples of all infants and their mothers at the family level. IF, infant feces; MF, maternal feces; BM, breast milk. *The unclassified bacteria at the family level. The **P_** in taxonomy labels indicate that the level of taxonomy is phylum. **(B)** The relative abundance of the biomarkers (at the genus level) in feces samples of 17 infants aged 0, 1, 2, 3, 4, 5, 6, 7, 8, 9, 11,12,13, 15, 20, 21, and 24 months (only one infant feces sample per month) and in feces and breast milk samples of all mothers (*n* = 25). The groups are ordered by hierarchical clustering of Bray–Curtis dissimilarities. *The unclassified bacteria at the genus level. #Other genera of Enterobacteriaceae. The biologically interpretable phenotypes (oxygen tolerance) are predicted by algorithm BugBase.

The extracted bacterial DNA was phylogenetically characterized by the 16S rRNA gene (V4–V5 regions) Illumina sequencing. A total of 1,620,470 high-quality reads were obtained. Reads were binned into 1,936 ASVs. The bacterial community was distinct between breast milk, maternal feces, and infant feces in both composition and diversity. In the breast milk and feces samples, the most abundant phylum was, respectively, *Proteobacteria* (average relative abundance: 46.5%) and *Firmicutes* (mothers: 60.2%, infants: 58.3%; Figure 1A). The two most frequently present families in the breast milk were *Enterobacteriaceae* (25.5%) and *Streptococcaceae* (19.3%). In the infant feces samples, *Streptococcaceae* (26.1%), *Lactobacillaceae* (16.3%), and *Enterobacteriaceae* (11.5%), were the three most dominant families. In the maternal feces, *Ruminococcaceae* (17.6%) and *Streptococcaceae* (9.2%) constituted the predominant families. *Pseudomonadaceae*, as a family consisting of mostly aerobic bacteria, has an average relative abundance (rel. ab.) in breast milk that was obviously higher (2.2%) than those in the feces (infants: 0.2%, mothers: 0.04%), whereas the average rel. ab. of *Ruminococcaceae*, as one of the most important anaerobic bacterial families, was lower in breast milk (<0.1%) than in feces (infants: 3.8%; mothers: 17.6%). The results of the adonis analysis based on Euclidean matrix calculated from microbial relative abundance at the family level (Supplementary Table 1) showed that the correlation between the microbial community structure (the family level) of breast milk and maternal BMI, feeding patterns, infants’ age, gender, and weight status was not significant (*R*^2^ < 0.2, *p* > 0.05); the microbial community structure of the maternal feces was only significantly correlated with the weight status (*R*^2^ = 0.23, *p* = 0.001), while that of infants’ feces was only significantly correlated with age (day; *R*^2^ = 0.13, *p* < 0.01).

We next used LEfSe to perform differentially abundant analysis at different taxonomic levels (from phylum to genus) between samples from three ecosystems (maternal feces, infant feces, and breast milk). Only the ASVs with relative abundance more than 0.01% were selected for the more accurate analysis. LEfSe analysis identified 109 differentially abundant genera and 198 differentially predominant families in three ecosystems (Supplementary Figure 1). From the cladogram that represents differentially abundant taxonomic level from phylum to genus level, it was evident that the breast milk harbored significantly more indicator taxa. In order to further observe the relationship of microbial composition of infants’ feces samples with that of samples from maternal feces and breast milk at different stages of lactation, we investigated the occurrence of biomarkers (LDA score > 3, *p* < 0.001) and dominant families in three ecosystems. Also, according to the results (Figure 1B), we found that feces microbiome of infants of younger age were more similar to breast milk microbiome (hierarchical clustering), which was represented by higher relative abundance of Enterobacteriaceae and *Citrobacter*, while the feces microbiome of infants with higher age were closer to those of mothers, mainly represented as higher relative abundance of *Prevotella* and *Catenibacterium*. It indicated that the temporal succession of the microbial community structure of the infants’ gut is actually a process by which the microbial composition similar to breast milk microbiome tends to be similar to the maternal gut microbiome.

**FIGURE 2:**
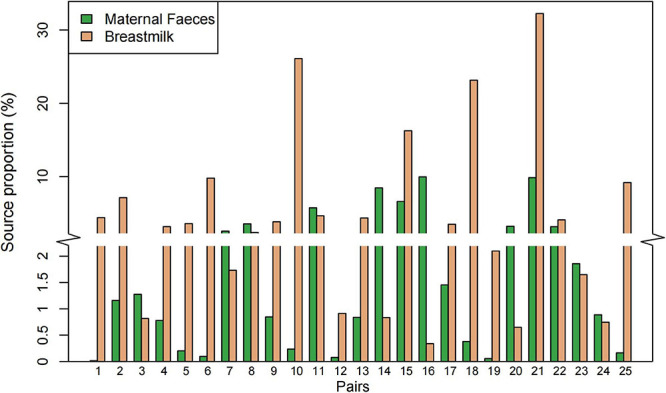
Proportion of the microbial sources at amplicon sequencing variants (ASV) level in infant feces using the FEAST model. The *y*-axis truncation value is 2.1–2.9.

Next, FEAST, a microbial sources tracking tool (Shenhav et al., 2019), was used to calculate the microbial source proportion of 25 infants’ feces, that from their mothers’ feces, and breast milk at ASV level. We found that the feces microbiome of the majority of infants (16/25) were more likely to be derived from their mothers’ breast milk than from their mother’s feces (Figure 2). The correlation between the ratio of source proportion of mother’s faces and breast milk and infants’ gender, weight status, and maternal obesity were not significant (*p* > 0.05; data not shown).

### Intra- and Intergroup Diversity Analysis

**FIGURE 3:**
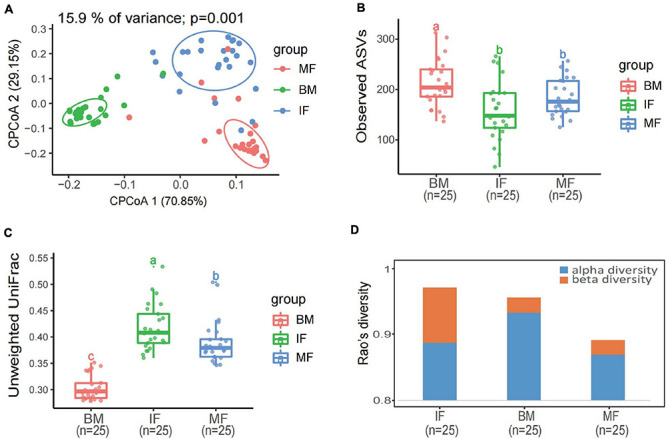
Diversity analysis of bacterial communities in infant feces, maternal feces, and breast milk samples. **(A)** Constrained principal coordinate analysis plot of Bray–Curtis distances between samples including three groups (15.9% of variance, *p* = 0.001; *n* = 75). Ellipses represent a 95% confidence interval around the cluster centroid. IF, infant feces; MF, maternal feces; BM, breast milk. **(B)** Box plot of the observed ASV index and **(C)** intragroup unweighted UniFrac distances calculated for maternal feces group (red), breast milk group (green), and infant’s feces group (blue) samples. Statistical analysis was performed using the permutational multivariate analysis of variance. **(D)** The α-diversity and β-diversity of three groups determined by Rao’s diversity decomposition at the ASVs level, considering both phylogeny and relative abundance.

A constrained principal coordinate analyses (CPCoA) based on the Bray–Curtis distance showed that the microbiota of breast milk, maternal feces, and infant feces, as expected, clustered separately, and the adonis test confirmed that the reported separation was significant (Figure 3A). The average microbiota profile obtained for breast milk was significantly more diverse (observed ASV index = 204 ± 109; Figure 3B) than both maternal and infant feces (176 ± 81 and 148 ± 118, respectively); interestingly, according to the unweighted UniFrac metric, the variability among breast milk samples was the lowest across all samples (0.30 ± 0.05; Kruskal–Wallis test *p* < 0.0001; Figure 3C). Meanwhile, the same result is presented in Figure 3D, which considers both phylogeny and relative abundance (α-diversity and β-diversity determined by Rao’s diversity decomposition at the ASVs level) (Rao, 1984). In other words, the breast milk ecosystem of the 25 enrolled mothers was more complex and less heterogeneous among samples (in terms of bacterial species) than the fecal ecosystem, suggesting that the bacteria from other ecosystems, except the maternal gut, also could be transferred to breast milk.

### Bacterial Groups With Statistical Differences

#### *Bifidobaterium* Profile Identified by 16S rRNA Gene Sequencing

It may be clear that seeding of early life microbiota with maternal microbes leaves a lasting imprint on the biology of infants. Considering the importance of *Bifidobacterium* as a health-promoting commensal of the gut microbiome in populations, it is necessary for us to delve deeply into the subdata of bifidobacteria in the whole dataset of mother and child pairs, rather than simply categorizing them into the “other” group in each sample as some reports have done. In the present study, in order to obtain more accurate taxonomic results at species level, the representative sequences of all 11 ASVs (which were identified as members of the *Bifidobaterium* genus) were extracted to homology search using NCBI BLAST^3^. Due to the limited resolving power of the 16S rRNA gene in the identification of different bacteria species, only three ASVs were clearly assigned into the *Bifidobacterium* species level: *Bifidobacterium adolescentis*, *B. pseudolongum*, and *B. bifidum*, respectively. The representative sequences of the remaining eight ASVs corresponded to four species groups (Query Coverage and Percent Identity were both 100%) of *Bifidobacterium* (each contains multiple closely related species recognized), namely, *B. breve/longum* (three ASVs), *B. pseudocatenulatum/kashiwanohense* (three ASVs), *B. adolescentis/faecale* (one ASV), and *B. angulatum/merycium* (one ASV). Overall, the average relative abundance of *Bifidobacterium* species groups varied depending on the types of samples, and their prevalence differed significantly among sample sets of three ecosystems. As shown in Table 2, the most predominant phylotype was the one belonging to the *B. breve/longum* group, the prevalence of which was 100% in infant feces. As expected, the average rel. ab of the *B. adolescentis* in maternal feces was higher than that in breast milk and infant feces, but its detection rate was only 72% in maternal feces, with seven maternal feces being negative. The *B. pseudocatenulatum/kashiwanohense* group was detected in approximately 80% of infant feces. The detection rate of *B. bifidum* in breast milk and infant feces was higher than in maternal feces. *B. pseudolongum* was detected in only four breast milk and undetected at any feces samples. Because the sequences that were annotated to match *Bifidobacterium* accounted for a very low proportion in the 16S rRNA gene datasets of all three types of samples, a limited number of ASVs were found to share among the three ecosystems.

**TABLE 2:** The relative abundance and detection rate of bifidobacteria group obtained by the National Center for Biotechnology Information (NCBI) comparison in the three ecosystems.

**Taxa**	**Average relative abundance**		
	**(detection rate) (%)**		
	**MF**	**BM**	**IF**
*Bifidobacterium bifidum*	0.003 (12)	0.053 (64)	0.104 (60)
*Bifidobacterium breve/longum*	0.107 (56)	0.283 (96)	1.834 (100)
*Bifidobacterium pseudocatenulatum/kashiwanohense*	0.028 (24)	0.011 (32)	0.358 (80)
*Bifidobacterium adolescentis*	0.031 (72)	0.019 (24)	0.017 (24)
*Bifidobacterium adolescentis/faecale*	0.057 (4)	0.054 (64)	0.003 (12)
*Bifidobacterium pseudolongum*	0 (0)	0.016 (16)	0 (0)
*Bifidobacterium angulatum/merycium*	0.002 (16)	0 (4)	0.004 (20)

#### *Bifidobacterium* Profile Identified by *gro*EL Gene Sequencing

In *gro*EL gene Illumina sequencing dataset, two samples (a maternal feces sample and an infant feces sample from a different mother–infant pair) with extremely low reads (<800) were removed. A total of 1,702,445 high-quality reads were obtained, and then were binned into ASVs (*n* = 376) according to the denoising (error correcting). As expected, the large majority of the recovered reads (90%) matched the DNA of the members of the genus *Bifidobacterium*. In order to accurately assess the association between maternal breast milk and fecal bifidobacterial community, our analysis was performed at the species level as much as possible. Consequently, multiple ASVs that hit the same nearest neighbor were identified as belonging to a specific species and/or subspecies regardless of the small sequence divergence. Taxonomic annotation showed that 376 ASVs were assigned to at least 13 members of *Bifidobacterium* that contain five well known subspecies, including *B. adolescentis* (counts of ASVs: 49), *B. angulatum* (34), *B. animalis* subsp. *animalis* (4), *B. animalis* ssp. *lactis* (4), *B. bifidum* (36), *B. breve* (16), *B. kashiwanohense* (85), *B. longum* ssp. *infantis* (58), *B. longum* ssp. *longum* (36), *B. pseudocatenulatum* (22), *B. pseudolongum* (8), *B. pseudolongum* ssp. *globosum* (6), and *B. ruminantium* (18). A neighbor-joining phylogenetic tree containing the representative sequences of all ASVs and closely related bifidobacterial taxon was constructed (Supplementary Figure 2).

**FIGURE 4:**
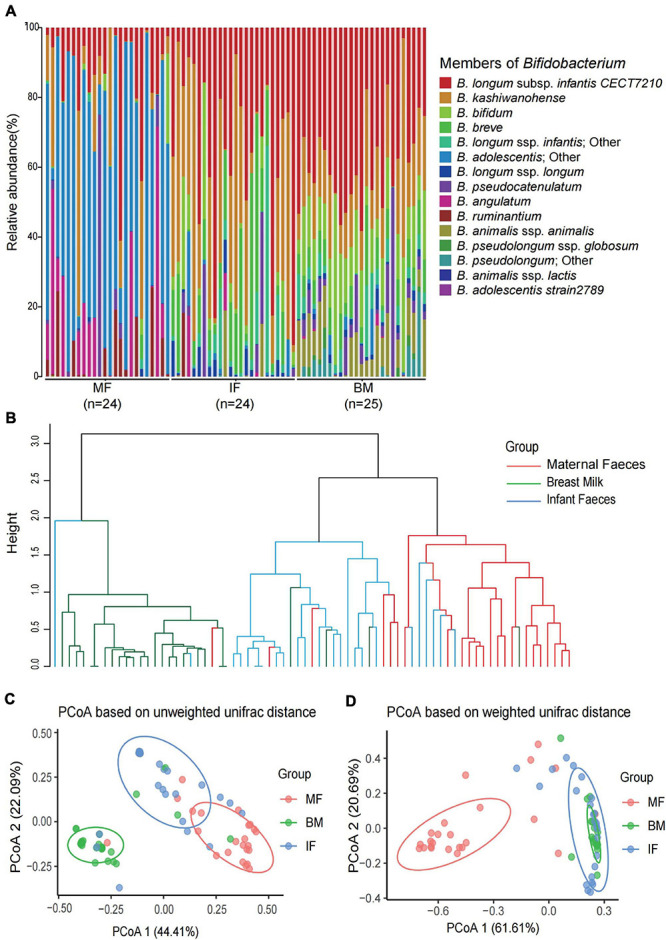
The composition and diversity of *Bifidobacterium* phylotypes in each infant feces, maternal feces, and breast milk samples. **(A)** The composition of *Bifidobacterium* phylotypes in each infant feces, maternal feces, and breast milk samples. **(B)** The hierarchical clustering based on unweighted UniFrac distance among the three ecosystems samples. Principal coordinate analysis based on unweighted UniFrac distances **(C)** and weighted UniFrac distances **(D)** among the three ecosystems. Ellipses represent a 95% confidence interval around the cluster centroid. In both PCoAs, first and second principal components (PCoA1 and PCoA2) were plotted. The significant difference of each two groups in panels **(C,D)** was evaluated by permutational multivariate analysis of variance. All *p*-values of each two groups are less than 0.001.

The bifidobacterial community profiles presented in all samples among three ecosystems are shown in Figure 4, which showed noticeable differences between sample groups in the composition, relative abundance, and diversity. In infant feces samples, the bifidobacterial community structure (composition and abundance) were more similar to that in breast milk samples (Figure 4A). However, the beta diversity analysis exhibited diametrically opposed results. As was shown in the result of hierarchical clustering and PCoA based on the unweighted UniFrac distance (ignored the abundance of all phylotypes in each sample), the bifidobacterial flora in the infant feces is more similar to that in the maternal feces even if there were significant differences between the three ecosystems (*p* < 0.001) (Figures 4B,C). The result of PCoA based on weighted UniFrac distance indicated that *Bifidobacterium* microbiome of maternal feces samples was more distinctive compared with that of breast milk and infant feces (Figure 4D).

**FIGURE 5:**
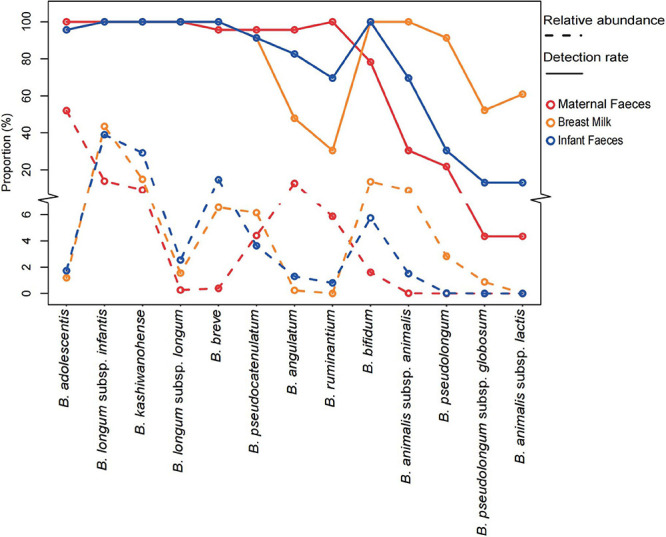
The relative abundance and detection rate of 13 *Bifidobacterium* species or subspecies in the three systems of 23 mother–infant pairs. The y-axis truncation value is 6.1–8.9.

Analysis of proportion abundances and detection rates (presence/absence), based on the whole *groEL* gene amplicons (Figure 5), shows that the dominant bifidobacteria taxon in breast milk and infant fecal samples were *B. longum* ssp. *infantis* (the average rel. ab. Of 43.5 and 39.0% in breast milk and infant feces, respectively, of the *groEL* gene amplicons of the genus *Bifidobacterium*) and *B. kashiwanohense* (14.8% in breast milk and 29.1% in infant feces), followed by *B. breve* and *B. longum* ssp. *longum.* On the contrary, the fecal samples from the mothers were dominated by *B. adolescentis* and *B. angulatum*, accounting for 52.0 and 12.6%, respectively, of the *groEL* gene amplicons of the genus *Bifidobacterium*. In contrast to maternal feces samples, the average rel. ab. of these two bifidobacterial species were very low in breast milk (1.2 and 0.2%) and infant feces (1.7 and 1.3%). Intriguingly, *B. bifidum*, considered as one of the infant-type bifidobacteria, was more abundant in the feces samples of mothers than that of infants. Another special bifidobacteria was *B. ruminantium* with the highest detection rates (100%) and lower relative abundance (0.06%) in maternal feces. However, its mean relative abundance in breast milk (<0.001%) and infant feces (0.8%) was significantly decreased, and no *B. ruminantium* ASV was detected in quite a few samples, especially in breast milk samples (detection rates in breast milk and infant feces: 30.4 and 70.0%).

#### The Co-occurrence of *Bifidobacterium* Phylotype in Mother–Breast Milk–Infant Triads Based on *gro*EL Gene

**FIGURE 6:**
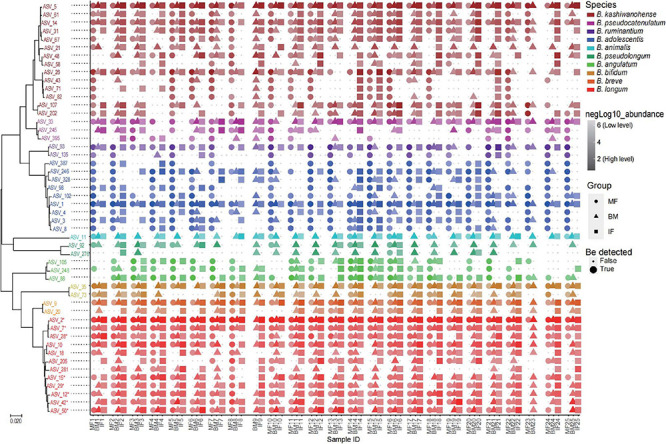
Phylogenetic tree of the 50 dominant ASVs and their co-occurrence in samples from 25 mother–breast milk–infant triads. The color of the text in the tree represents different bifidobacteria species. The color gradient of symbols represents the level of logarithm of relative abundance (the darker the color, the higher the relative abundance), the shapes represent different ecosystems, and the size is the logical value of whether the ASV can be detected in the sample. *B. longum subsp. *infantis*.

To get a better view of the bifidobacterial co-occurrence and association between mother–infant pairs, the detection rates (presence/absence) and average rel. ab. of each *Bifidobacterium* phylotype and its corresponding ASVs in breast milk and feces samples were analyzed deeply in mother–breast milk–infant triads (23 pairs with three ecosystem data simultaneously). Of 13 *Bifidobacterium* phylotypes (species or subspecies), 7 phylotypes, namely, *B. adolescentis*, *B. kashiwanohense*, *B. longum* ssp. *infantis*, *B. bifidum*, *B. breve*, *B. longum* ssp. *longum*, and *B. pseudocatenulatum*, were identified to be universally distributed in more than 90% samples of three ecosystems, with the exception of *B. bifidum* whose prevalence in the maternal feces was 78%, implying that they are shared nearly by all 23 mother–infant pairs analyzed (Table 3). ASV with an average rel. ab. greater than 1% in any one ecosystem was defined as dominant ASV, and 50 dominant ASVs were obtained. Among these 50 dominant ASVs, the co-occurrence of different ASVs belonging to the same *Bifidobacterium* phylotype was not exactly the same in infant–milk–mother triads (Figure 6). In these dominant ASVs, 14 ASVs were annotated as *B. kashiwanohense*, and they could be clustered into three clusters by a phylogenetic tree. Among them, the cluster composed of ASV_26, ASV_43, ASV_71, and ASV_82 had lower detection rates in breast milk samples than in feces samples; on the contrary, the detection rate of ASV_21 in breast milk was higher. It is worth noting that there were nine dominant ASVs annotated as *B. adolescentis*, but only one ASV has a high co-occurrence rate in infant–milk–mother triads (21/23). Moreover, out of a total of 49 ASVs, assigned to *B. adolescentis*, 18 ASVs were detected only in maternal feces samples in more than half of mother–infant pairs (more than 11 pairs) (Supplementary Table 2). In contrast, of the eight dominant ASVs annotated as *B. longum* ssp. *infantis*, six ASVs were co-occurrence in more than half of the infant–milk–mother triads of mother–infant pairs. Moreover, *B. ruminantium* and *B. angulatum* presented in almost all maternal feces samples, but were absent in more than half of the breast milk samples. In 11 mother–infant pairs, *B. ruminantium* was concurrently detected in the feces samples of infants and their mothers, but was not detected in the corresponding breast milk samples. Interestingly, we found that among the dominant ASVs annotated as *B. ruminantium*, if they can be detected in the infant feces sample, they can also be detected in feces samples of their mothers. In addition, the detection frequency of *B. animalis* and *B. pseudolongum* in breast milk samples (100 and 91.3%) was significantly higher than that in the feces samples of mothers (30.4 and 21.7%) and infants (30.4 and 69.6%). In terms of the same mother–infant pair, the dominant ASV (ASV_11), annotated as *B. animalis* ssp. *Animalis*, was concurrently detected in infants’ feces samples and their mothers’ breast milk samples of 16 mother–infant dyads, but present only in the feces samples of the corresponding five mothers.

**TABLE 3:** The occurrence frequency of 13 *Bifidobacterium* species or subspecies in samples from 23 mother–infant pairs.

**Taxa**	**Mother–infant pairs**				
	MF and BM*	MF and IF*	BM and IF*	MF and BM and IF**	All not***
*B. adolescentis*	1	1	0	20	1
*B. angulatum*	0	8	2	10	0
*Bifidobacterium ruminantium*	4	11	0	4	0
*B. pseudolongum* ssp. *globosum*	0	1	1	0	9
*B. pseudolongum*	3	0	5	2	2
*Bifidobacterium animalis* ssp. *animalis*	2	0	11	5	0
*B. pseudocatenulatum*	1	1	1	19	0
*B. breve*	0	0	1	22	0
*B. bifidum*	0	0	5	18	0
*B. kashiwanohense*	0	0	0	23	0
*B. longum* ssp. *infantis*	0	0	0	23	0
*B. longum* ssp. *longum*	0	0	0	23	0
*B. animalis* ssp. *lactis*	0	2	2	0	8

*Can only be detected in two ecosystems simultaneously.

**Can be detected in three ecosystems simultaneously.

***Cannot be detected in any of three ecosystems.

**FIGURE 7:**
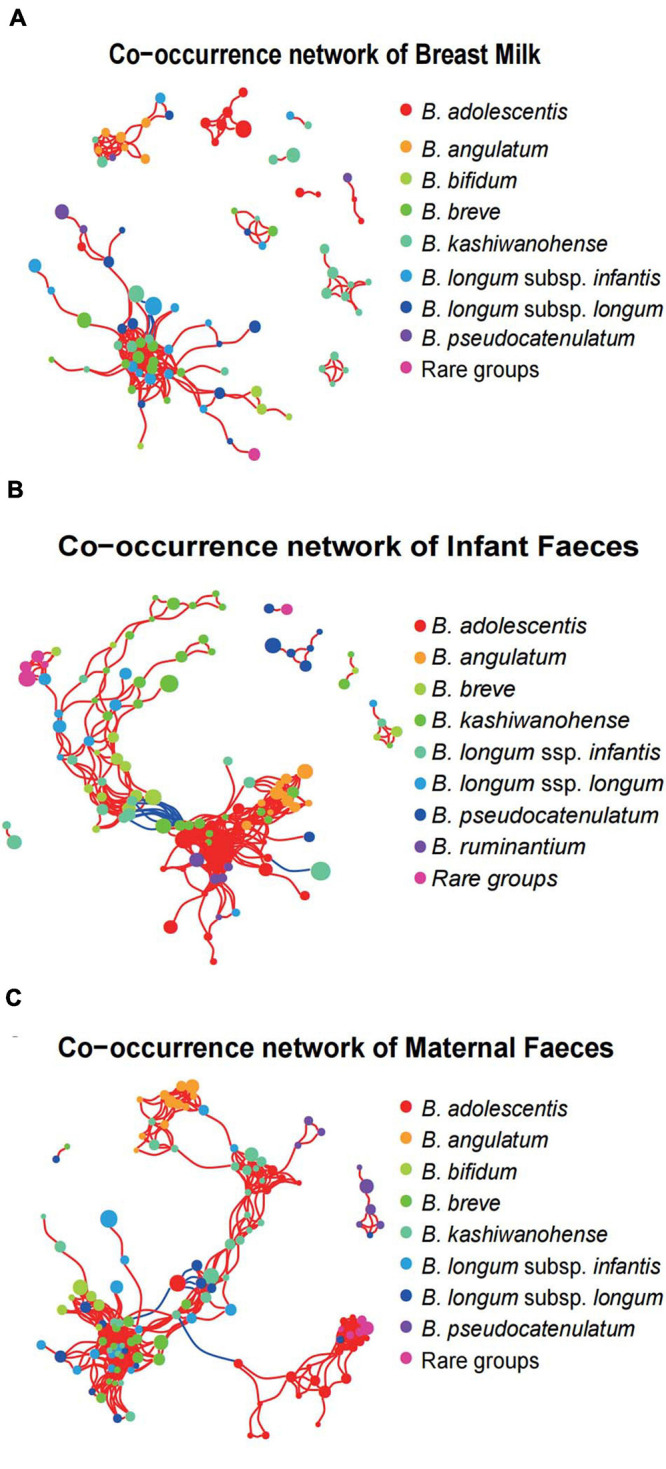
Bifidobacterial co-occurrence networks in the three systems. Co-occurrence networks of *Bifidobacterium* phylotypes in breast milk **(A)**, infant feces **(B)**, and maternal feces **(C)**. Each node in the network indicates an ASV, and the color represents bifidobacterial phylotypes. Each co-occurring pair among bacterial populations has an absolute Spearman rank correlation above 0.7 [red line: positive correlation (*R* > 0.70); blue line: negative (blue) correlation (*R* < –0.70)] with an FDR-corrected significance level under 0.01.

To investigate the concordance of microbial co-occurrence between the three ecosystems, we next assessed the correlations between ASVs, which were classified into eight common *Bifidobacterium* species (or subspecies) and rare groups. Approximately 87–122 ASVs (nodes) and 219–524 connections (edges) were retained in the co-occurrence networks across all ecosystems (Figure 7). The components and topographies of the networks of the breast milk group were significantly different from those of the feces sample groups, and the most remarkable difference was the connections of adult- to infant-type bifidobacteria. The topographies of the network of the breast milk group (Figure 7A) were relatively simple with a connection index of 0.059, compared with 0.062 and 0.071, respectively, for the infant feces (Figure 7B) and maternal feces groups (Figure 7C). The connections of adult-type bifidobacteria (*B. adolescentis* and *B. angulatum*) showed low frequencies of co-occurrence across breast milk samples with other bifidobateria, especially the so-called infant-type bifidobacterial species. For example, the ASVs annotated to *B. adolescentis* in the network of breast milk samples emerged as a separate cluster and had no co-occurrence with ASVs of other bifidobacteria (Figure 7A). In the network of infant fecal *Bifidobacterium* microbiota, a cluster with tight positive correlation was formed by typical infant-type bifidobacteria, *B. breve*, and *B. longum* ssp. *infantis*, along with *B. longum* ssp. *longum*, while adult-type bifidobacteria (*B. adolescentis* and *B. angulatum*) formed another cluster with *B. ruminantium* and a small number of ASVs annotated to *B. kashiwanohense*. It is noted especially that these two major clusters showed a significant negative correlation by multiple connections (Figure 7B). In the bifidobacterial co-occurrence network of maternal feces, one obvious characteristic is that there is significant negative correlation between one *B. adolescentis* ASV with the highest average relative abundance, and multiple ASVs belong to the *B. kashiwanohense* and *B. longum* ssp. *longum.* In addition, the vast majority of adult-type bifidobacterial ASVs (*B. adolescentis* and *B. angulatum*) had very few connections with other ASVs, although some *B. angulatum* ASVs were positively correlated with one *B. longum* ssp. *infantis* ASV (Figure 7C).

## Discussion

There is evidence that breast milk acts as an intermediary for the transfer of functionally important bacteria from mother to infant (Lyons et al., 2020). In particular, the interest in the gut symbiotic members of *Lactobacillus* and *Bifidobacterium* that can colonize the infant gut has grown significantly, mainly because their presence has been associated with a healthy microbiota (Arboleya et al., 2016; Ojo-Okunola et al., 2019). Breast milk is an important source of *Bifidobacterium* for the newborn gut (Oh et al., 2016). Due to the fact that total bacterial loads of breast milk are five to seven orders of magnitude lower than in feces samples (Fernandez et al., 2013), the amount of bifidobacteria in breast milk samples is very low compared with feces samples, especially the feces samples of infants. As a result, most studies based on culture techniques have reported very inconsistent results regarding the number and combination of *Bifidobacterium* phylotypes in breast milks within a specific population or across cohorts (Martin et al., 2009; Jost et al., 2013; Soto et al., 2014; Milani et al., 2015; Damaceno et al., 2017). However, most studies on vertical transmission of *Bifidobacterium* strains between the mother infant pair confirmed their co-occurrence among maternal intestine, breast milk, and the corresponding infant’s intestine. Nevertheless, such potential vertical transmission and the co-occurrence of *Bifidobacterium* is still only partially understood.

In the current study, the microbial composition of the three ecosystems was remarkably different: the abundance of aerobic bacteria (such as Pseudomonadaceae) in breast milk and that of anaerobic bacteria (such as Ruminococcaceae) in the maternal gut was significantly higher than another two ecosystems. This observation could be explained by the different conditions of the resident communities in the three ecosystems for pH, oxygen levels, and nutrients availability as reported also in earlier studies (Chen et al., 2018). However, within the cohort as a whole, *Bifidobacterium* spp. were detected at low relative abundances (mean relative abundances of 0.23, 0.44, and 2.32%) in the samples of the three ecosystems (maternal feces, breast milk, and neonatal feces). Based on very accurate and painstaking analyses of 16S rRNA gene dataset, our results showed that except for one maternal feces sample where no reads assigned to *Bifidobacterium* was observed, the presence of 16S rRNA reads assigned to *Bifidobacterium* could be identified in all breast milk and other feces samples, but its relative abundance was as low as 0.004% in a few breast milk and 0.02% in a few infant feces samples (data not shown). Previous studies showed that total bacteria concentration is lower in colostrum than in transitional and mature milk, with increasing levels of *Bifidobacterium* in breast milk over lactation time (Cabrera-Rubio et al., 2012; Khodayar-Pardo et al., 2014). In fact, the presence of *Bifidobacterium* was sporadically reported in a few colostrum samples or not at all (Hunt et al., 2011; Boix-Amoros et al., 2016; Drago et al., 2017; Chen et al., 2018). In our other study, in only about 5.4% of colostrum samples were retrieved 16S rRNA ASVs corresponding to *Bifidobacterium*, with <0.05% mean relative abundance (data not shown).

According to the published literature, most studies using high-throughput sequencing of 16S rRNA gene amplicon have reported conspicuous intercohort variation regarding the prevalence and relative abundance of *Bifidobacterium* in microbiome of breast milks and the infant gut, when compared with the stability of the adult gut microbiome (Murphy et al., 2017; Lackey et al., 2019; Moossavi et al., 2019). In the present study, it is curious that the relative abundance of *Bifidobaterium* (2.32%) in the microbiome of the infant gut was much lower than that in similar studies by other research groups, who reported that bifidobacteria achieved large proportions of the gut microbiota in the first few months after birth, ranging from 10 to 90% (Turroni et al., 2012; Lim et al., 2016; Duranti et al., 2017; Lundgren et al., 2018). The high abundance of bifidobacteria in the gut of breastfeeding infants was explained by the fact that host-derived glycans, like gut mucins and breast milk oligosaccharides in breast milk as specific growth substrates fertilize bifidobacterial growth, especially *B. longum* subsp. *infantis*, *B. bifidum*, and *B. breve* containing specific gene clusters encoding enzymes that are capable of hydrolyzing HMOs (Aakko et al., 2017; Turroni et al., 2017; Lawson et al., 2020). Thus, further research is needed to determine the reason for such a low relative abundance of *Bifidobacterium* in the intestinal tract of infants across our cohort, especially the association with breast milk oligosaccharides and diet and lifestyle, and environmental factors. In fact, some previous studies similarly reported that a proportion of infants targeting other populations have very low abundance or undetectable bifidobacteria as members of the fecal microbiota regardless of breast milk or formula feeding (Tannock et al., 2013, 2016). Duranti et al. (2017) reported very large interindividual variations for the relative abundance of *Bifidobacterium* in infant gut microbiota, with an abundance as low as 0.05%. In adults, bifidobacteria have been reported to commonly make up 1–10% of the gut microbiota (Arboleya et al., 2016). In our cohort, *Bifidobacterium* spp. were detected at low proportions (0.23%) in maternal feces samples. In fact, it was surprising that the complete absence of bifidobacteria in the Hadza gut microbiota is reported (Schnorr et al., 2014).

Given that determining the bacterial species with partial 16S rRNA sequences has to be taken with care, the representative sequences of all 11 *Bifidobaterium* ASVs in the 16S rRNA gene dataset were NCBI BLAST homology searched in order to obtain more accurate taxonomic results. Our results showed that they were annotated to seven species or species groups, of which the most predominant phylotype was the *B. breve/longum* group in breast milk and infant gut, with the prevalence being 100%. Although the average rel. ab of the *B. adolescentis* in maternal gut was higher than that in breast milk and infant gut, its prevalence was only 72% in the maternal gut, with seven maternal gut samples being negative. By contrast, the relative abundance of *Bifidobaterium* in the breast milk microbiome is similar to what has been reported in other studies. For example, in a study that targeted HBM samples collected between 6 and 10 weeks postpartum from lactating South African women, the average relative abundances of the genus *Bifidobacterium* was about 1.0%, yet with 28% of breast milk samples being free of bifidobacteria (Ojo-Okunola et al., 2019). In another study (Moossavi et al., 2019), NGS data based on 16S rRNA gene sequencing of V4 hypervariable region revealed that only 39% of breast milk samples contained *Bifidobacterium*, with mean relative abundance of 0.25 ± 0.98%. A recent study (Kumar et al., 2016) investigated the influence of geographical location on breast mature milk microbiota of healthy mothers, and the genus *Bifidobacterium* was found only in samples from South African women and not from Finland, Spain, and Beijing, China. Furthermore, in a study of 145 American women at approximately 6 weeks postpartum, mean relative abundances of the genus *Bifidobacterium* was about 0.65%. However, the relative abundances of *Bifidobacterium* were significantly different between breast milk microbiome types (Lundgren et al., 2018). In addition, a previous study in Taiwan and mainland China similarly reported that *B. longum* was the predominant bifidobacterial species with mean relative abundance of 0.3%, but its prevalence was only 62.4% (Li et al., 2017). Some studies have not even reported the presence and relative abundance of *Bifidobacterium* in breast milk during the first months postpartum (Browne et al., 2019), while other studies reported that *Bifidobacterium* spp. were detected at low proportions in human breast milk samples, but their prevalence was not described (Boix-Amoros et al., 2016; Chen et al., 2018; Meehan et al., 2018).

As described in all studies mentioned above, most studies on breast milk microbiome using the NGS of specific 16S variable gene regions reported only their results at the taxonomic level of genus. Particularly, the pitfalls inherent to 16S subregions, such as V3–V5, including the limited discriminating power among sequences belonging to the phylum Actinobacteria and the underrepresentation of rare OTUs with low abundance, may lead to skewed estimates of bifidobacterial community (Ellegaard and Engel, 2016; Johnson et al., 2019). Consequently, the choice of PCR primers can lead to underrepresentation of bifidobacterial community in 16S rRNA sequencing dataset, concealing the number and diversity of species- and strain-level sequence variants (Sim et al., 2012; Alcon-Giner et al., 2017).

In addition to the above reasons, another possible explanation for the underrepresentation of *Bifidobacterium*, which is in the downstream data analysis methods, some rare bacterial genus with very low abundance, including *Bifidobacterium*, which might be categorized into the “other” group in each sample, cannot be ruled out (Meehan et al., 2018). Particularly at the ASV level, taxa exhibiting such a low relative abundance (reads less than 10) are often dismissed in data analysis of high throughput for microbial community and diversity analysis, thereby leading to inconsistent results regarding the occurrence of bifidobacteria in mother–infant pairs (Chen et al., 2018). All of the above situations may be the reasons why there are always portions of the same set of samples from each of multiple independent cohort studies showing *Bifidobacterium* negative, despite using the same method.

A very interesting phenomenon was that the publications that investigated the bacteria vertical transfer from mother to infant have not reported the vertical transfer of *B. longum* subsp. *infantis* to date. The most likely explanation of this finding is that, in contrast to *B. longum* subsp. *longum*, which can be detected in the intestinal tracts of both adults and infants, the *B*. *longum* subsp. *infantis*, as a typical infant-type bifidobacterial phylotype is difficult to capture because it is a very low abundance taxon in the adult gut, even using metagenomic methods (Ward et al., 2013; Zhang et al., 2015; Vatanen et al., 2019). For example, only 10% of Finnish infants harbored *Bifidobacterium longum* subsp. *infantis*, whereas Russian infants commonly maintained a probiotic *Bifidobacterium bifidum* strain in infancy (Vatanen et al., 2019). More recently, the *groEL* gene proved to be a very effective target for the identification and quantification of *Bifidobacterium* spp. through high-throughput sequencing technologies or qPCR (Junick and Blaut, 2012; Hu et al., 2017). In our study, according to the denoising, taxonomic annotation showed that 376 *Bifidobaterium* ASVs in the *groEL* gene dataset were assigned to at least 13 well-known *Bifidobacterium* species or subspecies. Of them, seven phylotypes, including *B*. *longum* subsp. *infantis*, were identified to be universally distributed in all 23 mother–infant pairs analyzed. More remarkably, the so-called infant-type *Bifidobacterium* phylotype, such as *B. bifidum*, *B. breve*, and *B. longum* subsp. *Infantis*, was found to be present in the gut of all mothers, while *B. adolescentis*, a typical adult-type bifidobacterial phylotype, was almost detected in all infant feces and breast milk samples. Therefore, in terms of the occurrence and ecological distribution of human-associated bifidobacterial species, there should not be a very strict infant vs. adult subdivision. Our test results were obviously to support the hypothesis that breast milk as a seeding mediator inoculated the infant gut microbiome, regardless of the infant-type *Bifidobacterium* phylotype and adult-type bifidobacterial phylotype (Turroni et al., 2017). Of interest, *Bifidobaterium* ASV data obtained by the *groEL* gene sequences revealed relatively high intrasubject variability in *B. longum*, including *B. longum* ssp. *infantis* (58 ASVs) and *B. longum* ssp. *longum* (36 ASVs), compared with other common *Bifidobacterium* species, *B. bifidum* (36 ASVs), or *B. breve* (16 ASVs), which was consistent with the results reported in a recent study on strain-specific functional adaptation in the human gut microbiome during early life (Vatanen et al., 2019). Notably, comparison of the *groEL* gene-based ASVs identified in the data sets indicated the presence of identical ASVs in different sample pairs, implying that these identical sequences correspond to very closely related strains that presented in non-corresponding mother–infant dyads. Different ASVs of *B. kashiwanohense* showed different co-occurrence relationships in breast milk and feces samples, which may be caused by the bias of different strains to different ecosystems (Vatanen et al., 2019). Despite this genetic similarity, it can be determined that there may be great phenotypic variation between strains of the same species (homotypic strains) (Van Rossum et al., 2020). Further studies are needed to prove whether the phenotypic variation of strains in the mother-to-infant transmission process will be changed due to the ecosystem.

Moreover, there were very low relative proportion abundances of other six bifidobacterial taxa, whose prevalence (presence/absence) displayed both interindividual and sample set variations. According to the definition of microbial ecology, these *Bifidobacterium* phylotypes should belong to the rare taxa. However, thus far, little is known about the occurrence and the ecological relevance of rare *Bifidobacterium* phylotypes in human body environments. For example, in our cohort, rare *Bifidobacterium* phylotypes presented in the majority of maternal and infant gut, such as *B. ruminantium* and *B. angulatum*, with the highest detection rate (100%) and lower relative abundance (0.02%) in maternal gut, were not detected in quite a few breast milk samples. On the contrary, rare phylotypes like *B. animalis* and *B. pseudolongum*, showing almost 100% the detection frequency in breast milk samples, were absent in quite a few guts across all mother–infant pairs, especially in the mother gut. Among them, *B. animalis* ssp. *animalis* was concurrently detected in infants’ gut and their mothers’ breast milk of 16 mother–infant dyads, but presented only in the gut of the corresponding five mothers. In fact, *B. animalis* subsp. *lactis/animalis* and *B. pseudolongum*, which are frequently found in various animals, are scarcely encountered in the human intestinal tract, suggesting that they are not shared between mothers and their respective infant (Delcenserie et al., 2011; Lugli et al., 2019). Possibly, the probiotic endosymbiotic *Bifidobacterium* species are transmitted from mother to infant by direct vertical transmission during early life in most cases. However, another possibility that some oxygen-insensitive *Bifidobacterium* phylotypes with a cosmopolitan lifestyle (*B. animalis* and *B. adolescentis*) are indirectly transferred to breast milk and the infant’s intestinal tract through environmental contamination cannot be ruled out (Bondue and Delcenserie, 2015). Coincidentally, a study from an Argentine population described *B. animalis* subsp. *lactis* strains that originated from 16 breast milk samples (Zacarias et al., 2011). Besides, analogous to our result, a recent study also reported the presence of *B. pseudolongum* in the breast milk (Chen et al., 2018). Furthermore, our investigations are under way to clarify the importance and generality of the latter transmission route.

It is already known that there is a mutualistic cross-feeding or resource-sharing phenomenon in the bifidobacteria community of breastfed infant gut (Turroni et al., 2017). In the present study, we employed network-based analyses to elucidate the association and co-occurrence between *Bifidobacterium* phylotypes present in each of the three ecosystems of mother–breast milk–infant triads at the species or strain level (ASV). Strikingly, as shown in the network of infant gut, the major cluster where typical infant-type bifidobacterial ASVs intertwined with each other showed a significant negative correlation with a major cluster structured by adult-type bifidobacteria ASVs through multiple connections, indicating the presence of co-exclusion association. This result seems not to be in accordance with the reports by Turroni and cooperator (Turroni et al., 2016), who found that a strain of *B. adolescentis* exhibited mutalisitic cross-feeding behaviors when cocultured with *B. bifidum*, *B. breve*, and *B. longum* subsp. *infantis* strain, respectively. However, in the network of breast milk, the ASVs of different bifidobacterial phylotypes, including adult types, emerged as a separate cluster and displayed weak correlation with each other, suggesting that such ecological relationships might represent the random associations of bifidobacterial species in the poorly competitive ecosystem of breast milk. At present, our investigations are under way to clarify the association between maternal breast milk and infant fecal bifidobacterial profiles at the different stages of lactation, to pay close attention to the combination and longitudinal changes of *Bifidobacterium* phylotypes (species or strain level) between different ethnic groups during early life.

## Conclusion

By analyzing bacterial 16S rRNA gene datasets from the maternal stool, breast milk, and infant stool in a small yet very homogeneous cohort of 25 healthy Uyghur mother–infant pairs in Kashgar, Xinjiang, China, only three sets of ASVs could be clearly assigned into *B. adolescentis*, *B. pseudolongum*, and *B. bifidum*, respectively, whereas the remaining eight sets of ASVs corresponded to four *Bifidobacterium* species groups containing more than two closely related species. More remarkably, the *gro*EL gene proved to be a very effective mark gene for the depth resolution of *Bifidobacterium* community by high-throughput sequencing technology, allowing all *Bifidobaterium* ASVs to be assigned to at least 13 well-known *Bifidobacterium* species or subspecies. Among them, seven well-known *Bifidobacterium* phylotypes showed synchronism in 23 mother–infant pairs. However, several other rare bifidobacterial phylotypes, which were frequently encountered in animals, were found to display no correspondence of the presence between the three ecosystems of mother–infant pairs. Consequently, our test results were obviously to support the hypothesis that breast milk acts as an intermediary for the transfer of functionally important commensal bacteria from mother to infant, especially for endosymbiotic *Bifidobacterium* that can colonize the infant gut. In contrast, some oxygen-insensitive exogenous *Bifidobacterium* phylotypes with a cosmopolitan lifestyle may be indirectly transferred to breast milk and the infant’s intestinal tract through environmental contamination. Furthermore, the so-called infant-type *Bifidobacterium* phylotype, was found to be present in the gut of all mothers, while adult-type bifidobacterial phylotypes were almost detected in all infant gut and breast milk samples, indicating that there should not be a very strict infant vs. adult subdivision in terms of the occurrence and ecological distribution of human-associated bifidobacterial species.

## Data Availability Statement

The datasets presented in this study can be found in online repositories. The names of the repository/repositories and accession number(s) can be found below: www.ncbi.nlm.nih.gov/, BioProject ID PRJNA659245 and PRJNA702483.

## Ethics Statement

The studies involving human participants were reviewed and approved by Ethics Committee of the First Affiliated Hospital, Shihezi University School of Medicine. Written informed consent to participate in this study was provided by the participants’ legal guardian/next of kin.

## Author Contributions

YN and FT conceptualized the study and acquired the funding. WY and XZ conducted the experimental investigation. WY was in charge of the bioinformatics, statistics, figures, and wrote the original draft. YN wrote, reviewed, and edited the manuscript. BL collected the samples. All authors contributed to the article and approved the submitted version.

## Conflict of Interest

The authors declare that the research was conducted in the absence of any commercial or financial relationships that could be construed as a potential conflict of interest.

## Footnotes

1 http://www.personalbio.cn/

2 http://www.cpndb.ca

3 https://blast.ncbi.nlm.nih.gov/BlastAlign.cgi

## Supplementary Material

Supplementary Figure 1 Differentially abundant bacterial taxa between maternal feces (MF), breast milk (BM) and infant feces (IF) samples. **(A)** LEfSe comparison of microbiota in feces and breast milk samples. The genera listed in green describe breast milk, genera listed in blue describe infant feces, and genera listed in red describe maternal feces samples. Significant bacterial genera were determined by Kruskal–Wallis test (*P* < 0.05) with LDA score greater than 3. **(B)** Cladogram representation of differentially abundant bacterial taxonomic group detected using LEfSe. Different colors indicate the group in which clade was most abundant.

Supplementary Figure 2 Phylogenetic tree constructed based on the bifidobacterial ASV sequences. Outer circle color represents the phylogenetic strains.
