# Supplementary figures and images for: Association and Occurrence of Bifidobacterial Phylotypes Between Breast Milk and Fecal Microbiomes in Mother–Infant Dyads During the First 2 Years of Life
Source: Front Microbiol. 2021 Jun 7;12:669442. doi: 10.3389/fmicb.2021.669442 (PMC8215152; doi:10.3389/fmicb.2021.669442)

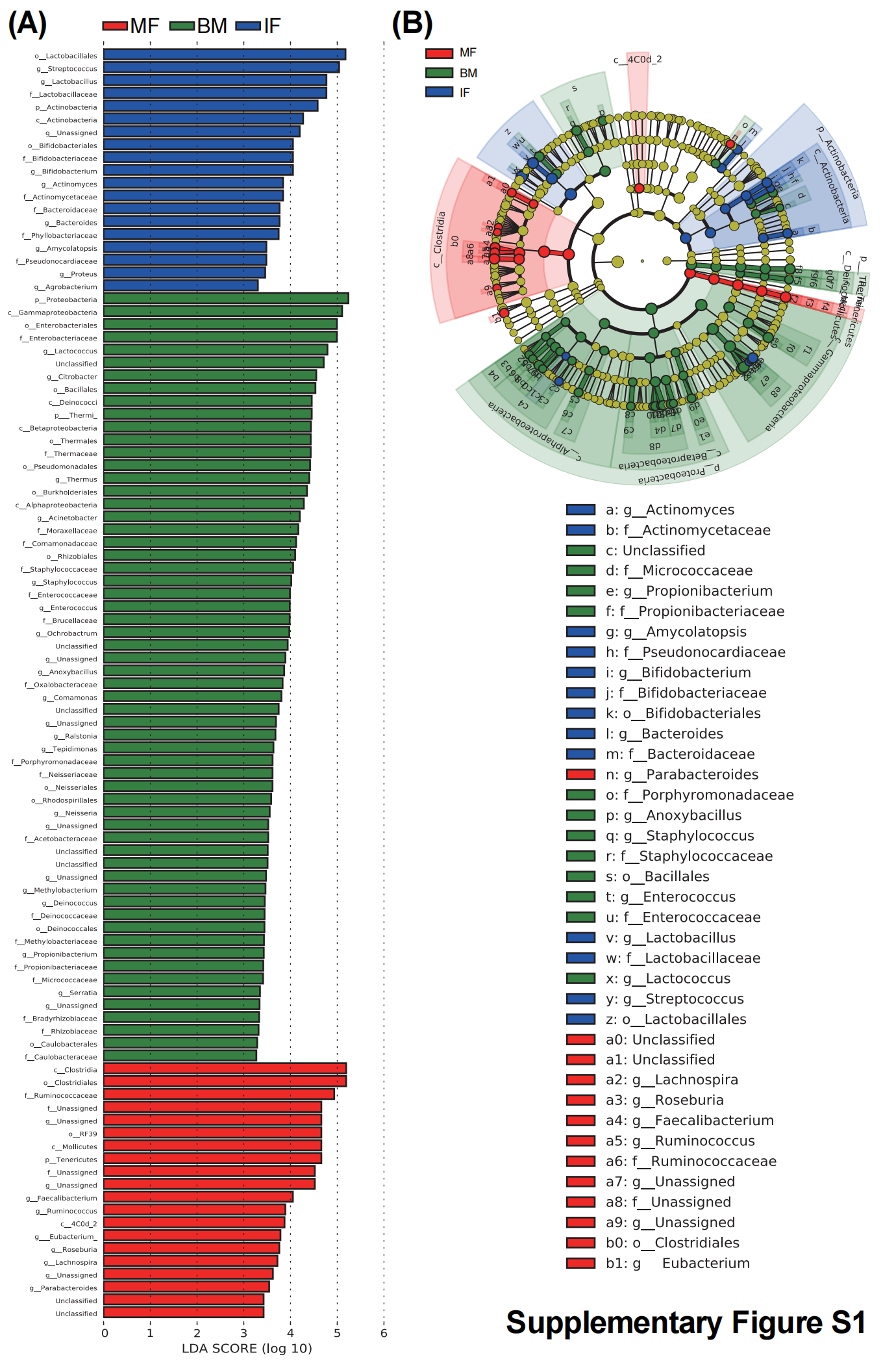

Supplement: Supplementary Figure 1 — Differentially abundant bacterial taxa between maternal feces (MF), breast milk (BM) and infant feces (IF) samples. (A) LEfSe comparison of microbiota in feces and breast milk samples. The genera listed in green describe breast milk, genera listed in blue describe infant feces, and genera listed in red describe maternal feces samples. Significant bacterial genera were determined by Kruskal–Wallis test (P < 0.05) with LDA score greater than 3. (B) Cladogram representation of differentially abundant bacterial taxonomic group detected using LEfSe. Different colors indicate the group in which clade was most abundant. [file Image_1.tif]

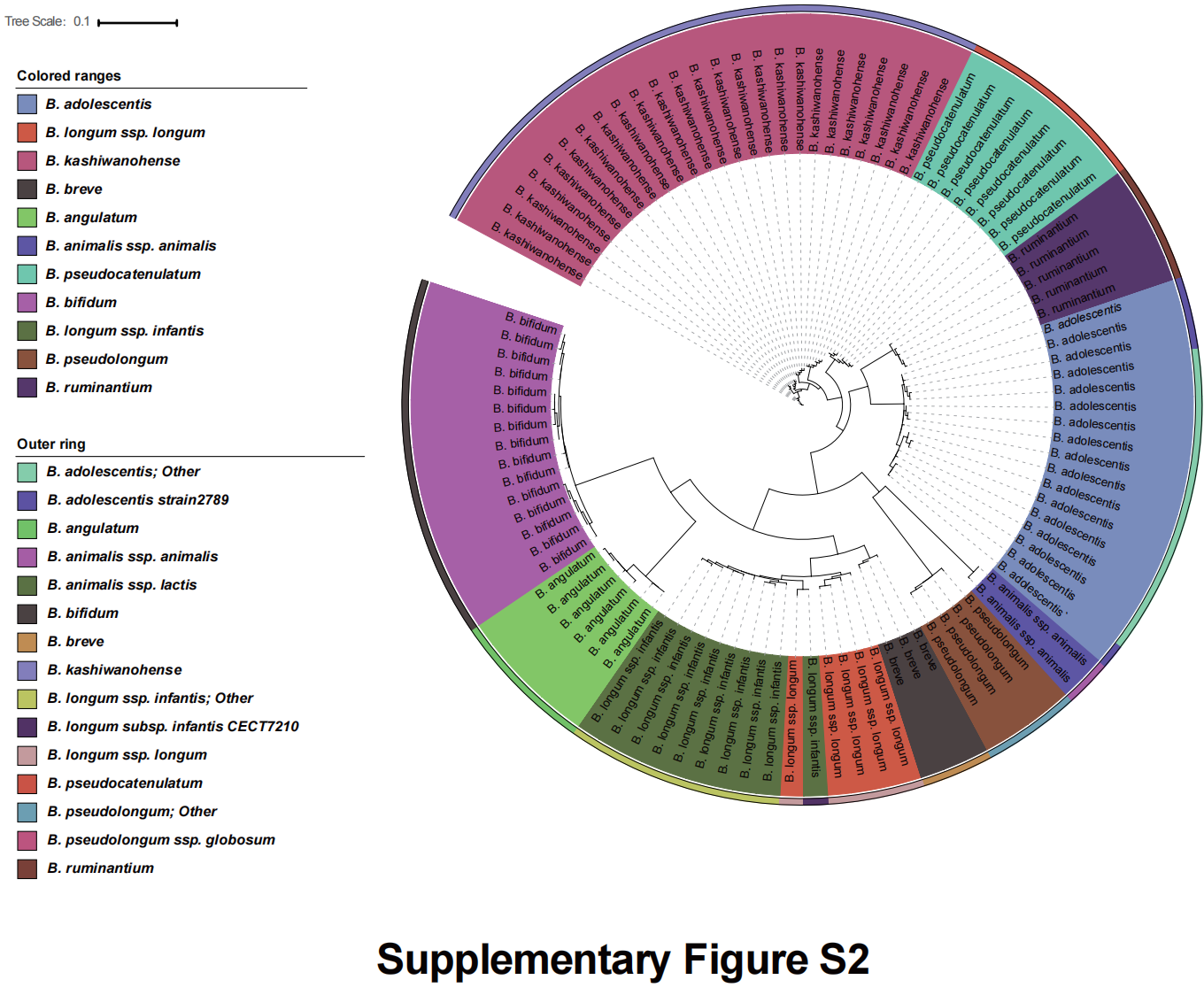

Supplement: Supplementary Figure 2 — Phylogenetic tree constructed based on the bifidobacterial ASV sequences. Outer circle color represents the phylogenetic strains. [file Image_2.tif]
