# Supplementary material for: Association and Occurrence of Bifidobacterial Phylotypes Between Breast Milk and Fecal Microbiomes in Mother–Infant Dyads During the First 2 Years of Life
Source: Front Microbiol. 2021 Jun 7;12:669442. doi: 10.3389/fmicb.2021.669442 (PMC8215152; doi:10.3389/fmicb.2021.669442)
Supplement: Supplementary file 3 [file Table_1.docx]

| **Supplementary Table S1 Adonis analysis based on Euclidean matrix** | | | | | | | | |
| --- | --- | --- | --- | --- | --- | --- | --- | --- |
| **Factors** | **BM** | |  | **MF** | |  | **IF** | |
|  | **R^2^** | **Pr(>F)** |  | **R^2^** | **Pr(>F)** |  | **R^2^** | **Pr(>F)** |
| Infant age (day) | 0.023 | 0.899 |  | 0.032 | 0.527 |  | 0.126 | 0.008 |
| Infant age (week) | 0.037 | 0.554 |  | 0.028 | 0.645 |  | 0.047 | 0.465 |
| Infant's gender | 0.062 | 0.153 |  | 0.059 | 0.084 |  | 0.041 | 0.537 |
| Infant's weight status | 0.185 | 0.365 |  | 0.184 | 0.148 |  | 0.139 | 0.854 |
| BMI of mother | 0.056 | 0.224 |  | 0.059 | 0.072 |  | 0.022 | 0.892 |
| Maternal weight status | 0.124 | 0.5 |  | 0.235 | 0.001 |  | 0.105 | 0.792 |
| Feeding patterns | 0.081 | 0.074 |  | 0.040 | 0.343 |  | 0.024 | 0.869 |
